# Microbiology research at the systems biology and bioinformatics - 2019 (SBB-2019) school

**DOI:** 10.1186/s12866-020-02038-5

**Published:** 2020-11-24

**Authors:** Yuriy L. Orlov, Alexander N. Ignatov, Elvira R. Galieva, Oxana B. Dobrovolskaya

**Affiliations:** 1 The Digital Health Institute, I.M. Sechenov First Moscow State Medical University of the Ministry of Health of the Russian Federation (Sechenov University), 119146 Moscow, Russia; 2 Novosibirsk State University, 630090 Novosibirsk, Russia; 3 Agrarian and Technological Institute, Peoples’ Friendship University of Russia (RUDN University), 117198 Moscow, Russia; 4 Institute of Cytology and Genetics SB RAS, 630090 Novosibirsk, Russia; 5 PhytoEngineering R&D Centre, 141880 Moscow Region Moscow, Russia

This Special Issue of BMC Microbiology “Systems Biology and Bioinformatics” presents the materials discussed at the 11-th Young Scientists School “Systems Biology and Bioinformatics”-2019 (SBB-2019) in Novosibirsk, Russia (http://conf.bionet.nsc.ru/sbb2019/en/). These Young Scientists’ Schools on bioinformatics have been organized every year since 2008 by the Institute of Cytology and Genetics of the Siberian Branch of the Russian Academy of Sciences and Novosibirsk State University [1, 2]. To accompany this Special Issue on microbiology, parallel special journal issues in the fields of genomics, bioinformatics, and medical genomics were published as a part of SBB-2019 series in BioMed Central journals: BMC Genomics, BMC Medical Genomics, BMC Genetics, BMC Medical Genetics, and BMC Bioinformatics (https://bmcbioinformatics.biomedcentral.com/articles/supplements/volume-21-supplement-11). The SBB Schools in Novosibirsk are satellite training meetings for young scientists and PhD students, which are organized either as satellite events for BGRS\SB (Bioinformatics of Genome Regulation and Structure \ Systems Biology) conferences series [3, 4] or as independent events [2, 5]. The Schools were accompanied and complemented by the publication of special journal issues in BMC Microbiology [6] and other BioMed Central journals [3, 4].

This special issue contains the study by S.E. Peltek and co-authors [7] describing microbial life habitats in unique volcano caldera environment.

The caldera of the Uzon Volcano (Kamchatka Peninsula, Russia) is a region with active hydrothermal activity, which contains outlets of unique natural hydrothermal petroleum [8] with distinct microbiota. Hydrothermal petroleum is the oil found in natural outlets within active hydrothermal fields [8, 9]. According to carbon ^14^C dating, hydrothermal petroleum from various regions of the Earth is modern in geological scale, with the oldest sample being 29,000 years old [10]. The Uzon petroleum is the youngest on Earth, with the initial time estimate at 1000 years, and later found to be only 50 years old [11].

The composition of the Uzon oil was investigated in several studies [12]. Uzon oil has a unique make-up, with low proportion of heavy fractions and relatively high content of saturated hydrocarbons [11]. Correspondingly, the microbial communities of the “oil site” have diverse composition profiles, living at almost boiling temperatures (up to 97 °C), significant oscillations of pH, and high content of sulfides, arsenic, antimony, and mercury in water.

In this journal issue, Peltek et al. [7] analyzed the composition, structure, unique genetic features and the metabolic pathways of the microbial communities at the oil site. The authors present evidence of diverse metabolic pathways of hydrocarbon degradation by microorganisms being operational within the local microbiota. Interestingly, the authors found statistically significant relationships between geochemical parameters, taxonomic composition and the completeness of metabolic pathways [7]. Metabolic pathways of hydrocarbon oxidation were found to prevail in the studied communities, which corroborated the hypothesis on abiogenic synthesis of Uzon hydrothermal petroleum.

In previous research, the majority of the studied oil sites contained representatives of *Actinomycetales* (Actinobacteria). Typically, the geochemical parameters defined the structure and metabolic potential of microbial communities. This is confirmed by the genome sequence of *Anoxybacillus flavithermus* KU2–6-11 isolated from hot-spring in Uzon caldera, which was published recently [13]. Other prior work includes the microbial community analysis of the Uzon caldera springs, which was presented earlier at the BMC special journal issues accompanying a previous BGRS conference in 2014 [14]. The same researchgroup, A.V. Bryanskaya et al., published description of environmental factors for the composition of microbial communities of saline lakes in BMC Microbiology special issue [15]. While this journal issue has been in preparation, novel thermophilic *Aeribacillus* bacteriophage AP45 was isolated from the Kamchatka [16] as well as a thermophilic bacterium *Geobacillus icigianus* [17].

Going back to the BGRS post-conference issues, we would like to note fundamental systems biology work by late Dr. Vitaly A. Likhoshvai on the mathematical modeling of metabolic systems in *Escherichia coli* cells, which was published in BMC Microbiology [6]. This research showed that the modeling of relevant environmental factors can increase a heuristic value of a genomic study of the microbial communities. The manuscript by V.A.Likhoshvai, which has been finalized by his co-authors, was published in the parallel BMC Bioinformatics special issue after SBB-2019 School [18]. Here it is also appropriate to refer to the recent publication by V.A. Likhoshvai on dynamical model of non-inherited antibiotic tolerance of microorganisms [19].

We aim to support international exchanges and education in bioinformatics and systems biology in the forms of the Schools for young scientists and make new results open keeping traditions of the BGRS conference series [1, 2, 5, 20].
